# Head of the Syrian Hamster (*Mesocricetus auratus*): Planar Anatomy with Correlative Micro-Computed Tomography and Magnetic Resonance Imaging

**DOI:** 10.3390/ani16111629

**Published:** 2026-05-27

**Authors:** Jamal Nourinezhad, Sadaf Ansari, Abdolvahed Moarabi, Mohammad Ghasem Hanafi, Maciej Janeczek

**Affiliations:** 1 Division of Anatomy and Embryology, Department of Basic Sciences, Faculty of Veterinary Medicine, Shahid Chamran University of Ahvaz, Ahvaz 61357-83151, Iran; 2 Faculty of Veterinary Medicine, Shahid Chamran University of Ahvaz, Ahvaz 61357-83151, Iran; 3 Division of Radiology, Department of Clinical Sciences, Faculty of Veterinary Medicine, Shahid Chamran University of Ahvaz, Ahvaz 61357-83151, Iran; 4 Department of Radiology, Ahvaz Jundishapur University of Medical Sciences, Ahvaz 61357-15753, Iran; 5 Division of Animal Anatomy, Department of Biostructure and Animal Physiology, Faculty of Veterinary Medicine, Wroclaw University of Environmental and Life Sciences, 51-631 Wrocław, Poland

**Keywords:** cross-sectional anatomy, macroscopic anatomy, mouth, rodent, small exotic mammals, stomatology, teeth, tomographic imaging

## Abstract

Syrian hamsters (SHs) are widely used in research and as pets. However, their head anatomy has not yet been evaluated using sectional anatomy and imaging despite their unique features, which are important for studying ischemia–reperfusion injury, cancer, oral tumors, and common stomatognathic and ocular conditions. This study was conducted to correlate the planar anatomy of the heads of eight healthy male and female SHs with micro-CT and MRI images to establish a descriptive, imaging-based anatomical reference. Clinically important head structures observed in transverse, dorsal, and sagittal anatomical sections were correspondingly identified on micro-CT and/or MRI images. In SHs, head micro-CT was shown to be particularly effective for visualizing mineralized structures (e.g., dental and osseous tissues) and air-filled cavities (e.g., the ear canal and tympanic bulla), whereas MRI was demonstrated to provide superior assessment of soft tissues, including the brain, vertebral canal and spinal cord, musculature, intervertebral disks, major salivary glands, eye, and harderian and extraorbital lacrimal glands. The present investigation provides a descriptive and imaging-based anatomical reference of the SH head by integrating anatomical sections, in situ topographical anatomy, and dry-skull photographs with micro-CT and MRI datasets, thereby serving as a foundational resource for the interpretation of cross-sectional imaging in both research and clinical contexts.

## Simple Summary

Syrian hamsters are widely used both as companion animals and as common laboratory species; however, detailed references describing their head sectional anatomy and diagnostic imaging remain limited. In this study, advanced imaging modalities—including micro-computed tomography and magnetic resonance imaging combined with anatomical sectioning—were employed to examine the cranial structures of the Syrian hamster. The resulting images and datasets provide a comprehensive reference for veterinarians, internal medicine specialists, surgeons, anatomists, radiologists, and other researchers.

## 1. Introduction

The Syrian hamster (*Mesocricetus auratus*) is a widely used research species, representing ~90% of laboratory hamsters. Its head has distinct anatomical and physiological features, most notably bilateral cheek pouches, which are valuable for studying ischemia–reperfusion injury, carcinogenesis, and oral tumors [1]. Moreover, Syrian hamsters are popular pets, with common head-related conditions affecting the stomatognathic and ocular systems [2,3,4,5].

Conventional radiography of the rodent and rabbit head is limited by complex anatomy, small size, and superimposition of osseous structures. Consequently, advanced tomographic modalities such as computed tomography (CT) and magnetic resonance imaging (MRI) are increasingly employed in research and veterinary practice [2]. By producing high-resolution, thin-slice images that eliminate structural overlap, these techniques enable detailed anatomical evaluation [6,7] and serve complementary diagnostic roles in the assessment of head pathologies in rodents and rabbits [3]. Among these modalities, CT is invaluable for evaluating head bones and subtle fractures and is considered the gold standard for diagnosing dental diseases in rodents and rabbits [3,4,8]. Additionally, micro-computed tomography (MCT) provides higher spatial resolution, faster scans, and cost-effectiveness, making it suitable for high-resolution imaging of small animals [9,10]. Originally for laboratory use, micro-CT is now applied in private veterinary practice and translational research, including studies of rodent inner ear pathologies [3,11].

In addition to CT and MCT, MRI is a non-invasive, computer-based imaging modality that does not use ionizing radiation and allows multiplanar image acquisition without subject repositioning. MRI is especially well-suited for soft tissue evaluation and is regarded as the modality of choice for imaging the central nervous system [12,13]. In pet rabbits and rodents, MRI is a very useful diagnostic tool for detecting soft tissue changes and assessing the presence and extent of abscesses [4]. Furthermore, MRI is extensively used in translational research involving rodent models of central nervous system, pituitary, and inner ear disorders [14,15,16,17].

Given these imaging capabilities, a thorough understanding of normal sectional head anatomy is therefore essential for the accurate interpretation of CT, micro-CT, and MRI images [18]. Accordingly, previous studies have described normal head anatomy using correlative CT and/or MRI alongside corresponding gross anatomical sections in cadaveric crested porcupines and rabbits, as well as in live guinea pigs [18,19,20,21,22]. In contrast, CT and MRI investigations lacking corresponding gross sections have been reported for the skull or brain of rabbits and capybaras [23,24,25,26,27,28]. Similarly, micro-CT studies without gross anatomical correlation have focused on osseous structures, musculature, the brain, and dentition in various species, including guinea pigs, mice, rats, gerbils, chinchillas, agoutis, capybaras, squirrels, and rabbits [29,30,31,32,33]. However, despite these advances, comparable correlative CT/micro-CT and/or MRI studies with matched anatomical sections remain unavailable for the Syrian hamster. Therefore, building on our previous investigations of gross and imaging anatomy in Syrian hamsters [34,35,36,37,38,39], the present study aimed to: (1) provide a color-enhanced morpho-topographic depiction of major head structures; (2) generate and analyze serial anatomical head sections for the first time; (3) establish a normative anatomical reference using sequential micro-CT imaging; (4) describe the normal MRI anatomy of the head; and (5) correlate gross anatomical sections with corresponding micro-CT and MRI images to achieve comprehensive anatomical validation.

## 2. Materials and Methods

### 2.1. Examined Animals

Eight healthy adult Syrian hamsters (mean ± SD body weight: 106.83 ± 2 g; age: 69 days) were included in this study. Age was estimated based on body weight according to Gad [40]. Health status was assessed via clinical history, physical examination, and whole-body radiographic and ultrasonographic screening (Z60 Vet, Shenzhen Mindray Animal Medical Technology Co., Ltd., Shenzhen, China), with no complications observed. Animals were provided by the Razi Vaccine and Serum Research Institute and the Center for Comparative and Experimental Medicine at Shiraz University of Medical Sciences and were maintained under controlled conditions (air-conditioned room, 12 h light/dark cycle) with ad libitum access to standard laboratory chow and water. Fasting was not required before radiographic, micro-CT, or MRI procedures [34,41,42]. For anesthesia, hamsters received intraperitoneal injections of ketamine (5 mL; Bremer Pharma GmbH, Warburg, Germany), acepromazine (1 mL; Alfasan, Woerden, The Netherlands), and xylazine (2.5 mL; Alfasan, The Netherlands), following previously described protocols [36,38].

### 2.2. Radiological Technique

Lateral and ventrodorsal head radiographic examinations were conducted using a portable X-ray (Dongmun-100P, Goyang-si, Republic of Korea) and a Prima CR radiology system (Model: CR-I392, Fujifilm, Minato City, Japan), following the positioning procedures of Silverman and Tell [41].

### 2.3. Micro-CT Technique

Head imaging was conducted with the animals positioned in ventral recumbency using an in vivo X-ray micro-CT scanner (LOTUS in Vivo, Behin Negareh Co., Tehran, Iran) at the Preclinical Core Facility of Tehran University of Medical Sciences. The scanning protocol employed an X-ray tube voltage of 80 kV, a current of 100 µA, a frame exposure time of 0.25 s, and 1.3× magnification. Each session required approximately 30 min, with reconstructed slice thickness set at 30 µm. Image acquisition was controlled by LOTUS-in vivo-ACQ, Version 1.0 software, and three-dimensional reconstruction was performed using the Feldkamp–Davis–Kress algorithm, producing DICOM datasets in sagittal, transverse, and dorsal planes. Window width and level adjustments were applied to enhance the quality of head visualization.

### 2.4. MRI Technique

Head imaging was conducted with a 3 Tesla clinical MRI scanner (Siemens MAGNETOM Prisma, Erlangen, Germany) using a 20-channel head coil, with the subjects positioned in ventral recumbency. MRI was performed using a T1-weighted spin-echo (SE) sequence with a slice thickness and interslice gap of 1.4 mm, a field strength of 1.5 T, three signal averages (NEX = 3), a bandwidth of 390 Hz/pixel, an echo train length of 2, a flip angle of 150°, and a matrix size of 512 × 400. Transverse images were acquired with a TR/TE of 1020/9.1 ms and a window level/window width of 314/669, while sagittal and dorsal images were obtained with a TR/TE of 2040/9.1 ms; the window level/window width was 357/777 for sagittal and 421/812 for dorsal scans. The slice thickness and voxel size were chosen to optimize image quality while minimizing scan time and reducing anesthesia-related risks [12,13,42]. Images were evaluated using Bee DICOM Viewer 2.5.1 (Beijing, China). The scan time for sagittal (dorsal) T1-weighted images was 10.4 min, whereas the scan time for transverse T1-weighted images was 16 min. The total acquisition time for a complete MRI examination of the head was approximately 37 min.

### 2.5. Anatomical Study

A total of eight Syrian hamsters were euthanized, with two used for in situ topographic anatomical studies and six for anatomical sectioning. For skull preparation, one of the specimens from the topographic anatomy group was macerated according to the method described by Hildebrand [43]. In the dissection photographs, a portion of a steel ruler was used as a scale bar, with the distance between the two lines set at 1 mm.

#### 2.5.1. In Situ Topographic Anatomy

After imaging, two Syrian hamsters were euthanized in accordance with AVMA guidelines [44], followed by dissection and photographic documentation using a Samsung Galaxy S24 Ultra (Suwon, Republic of Korea).

#### 2.5.2. Anatomical Sections

Six euthanized SHs were frozen in ventral recumbency at −70 °C and sectioned using an electronic bandsaw into ~5 mm transverse, sagittal, and dorsal slices, which were photographed. Anatomical structures were identified and labeled based on existing references [45,46] and the International Committee on Veterinary Gross Anatomical Nomenclature [47]. Correlations between micro-CT, MRI, and cadaveric slices were established to facilitate accurate labeling across imaging modalities.

## 3. Results

**Figure 1:**
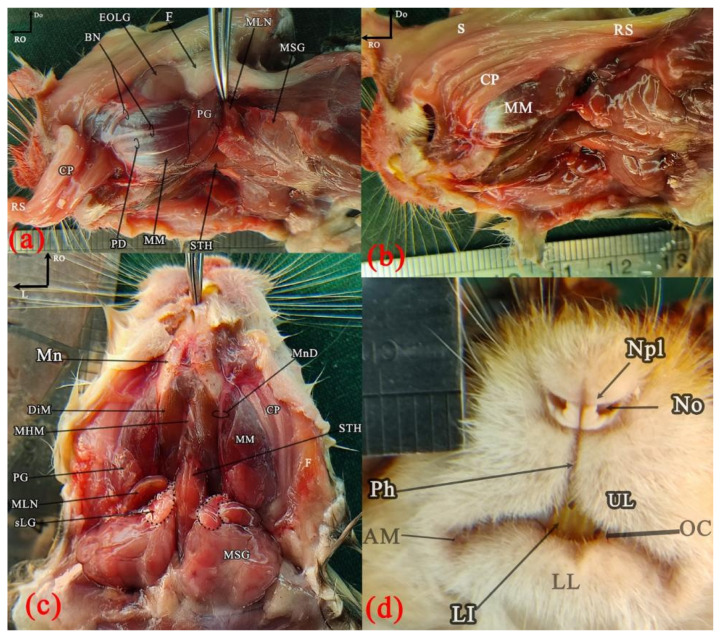
Superficial structures of a male fresh cadaver of the Syrian hamster head and cranial end of the neck: left lateral (**a**,**b**), ventral (**c**), and rostral (**d**) views. The retractor sacculi have been transacted caudally and displaced rostrally to expose the masseter muscle, parotid and mandibular salivary glands, and parotid duct (**a**). **AM**: Angle of the mouth, **BN**: Buccal nerve, **CP**: Cheek pouch, **DiM**: Digastric muscle, **EOLG**: Extraorbital lacrimal gland, **F**: Fat tissue, **LI**: Lower incisor teeth, **LL**: Lower lip, **MHM**: Mylohyoid muscle, **MLN**: Mandibular lymph node, **MM**: Masseter muscle, **Mn**: Mandible, **MnD:** Mandibular duct, **MSG**: Mandibular salivary gland, **No**: Nostril, **Npl**: Nasal palate, **OC**: Oral cleft, **PD**: Parotid duct, **PG**: Parotid salivary gland, **Ph**: Philtrum, **RS**: Retractor sacculi muscle, **S**: Skin, **STH**: Sternothyrohyoid muscle, **sLG**: Sublingual salivary gland, **UL**: Upper lip, **RO**: Rostral, **Do**: Dorsal, and **L**: Left. Scale: 1 mm.

**Figure 2:**
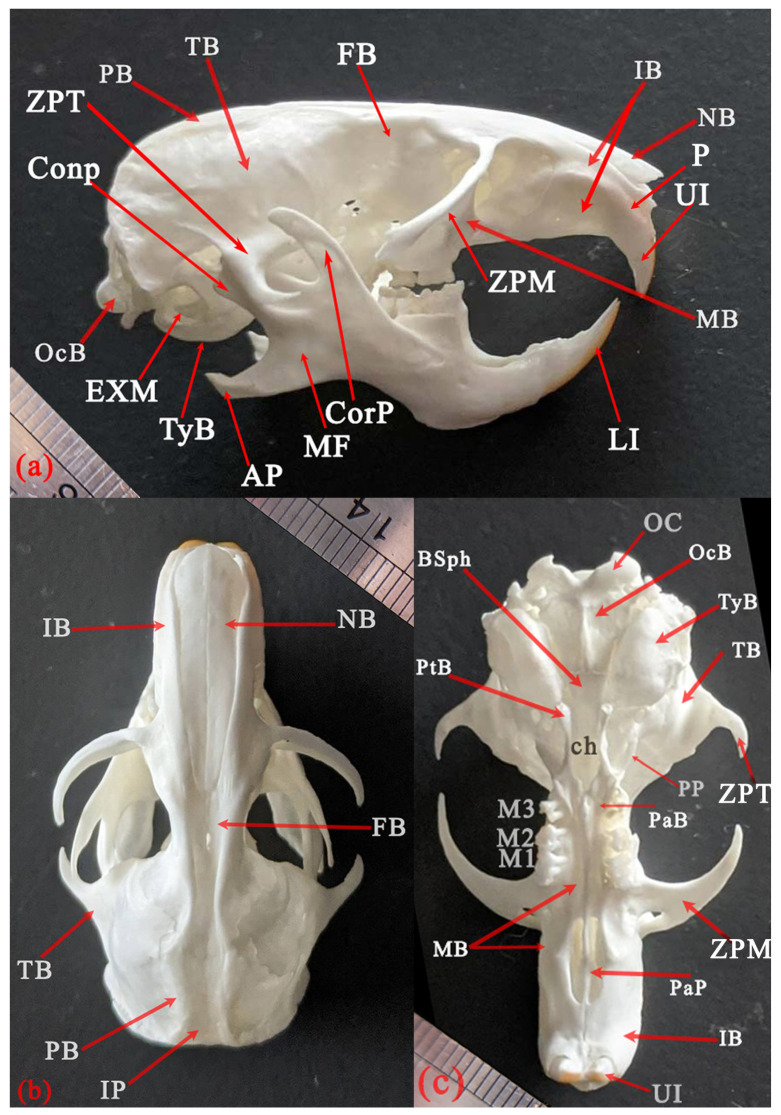
Skull of a Syrian hamster. Right lateral (**a**), dorsal (**b**), and ventral (**c**) views. **AP**: Angular process, **BSph**: Basisphenoid bone, **ch**: Choanae, **Conp**: Condylar process of mandible, **CorP**: Coronoid process of mandible, **EXM**: External acoustic meatus, **FB**: Frontal bone, **IB**: Incisive bone, **IP**: Interparietal bone, **MB**: Maxillary bone (palatine process in ventral view), **MF**: Masseteric fossa, **M 1–3**: First molar tooth to third molar tooth, **LI:** Lower incisor tooth, **NB**: Nasal bone, **OC**: Occipital condyle, **OcB**: Occipital bone (basilar part in ventral view), **P**: Prominence of alveolar crown of tooth, **PaB**: Horizontal palate of the palatine bone, **PaP**: Palatine process of incisive bone, **PB**: Parietal bone, **PP**: Vertical palate of the palatine bone, **PtB**: Pterygoid bone, **TB**: Temporal bone, **TyB**: Tympanic bulla, UI: Upper incisor, **ZPM**: Zygomatic process of maxilla, and **ZPT**: Zygomatic process of temporal; Scale: 1 mm.

**Figure 3:**
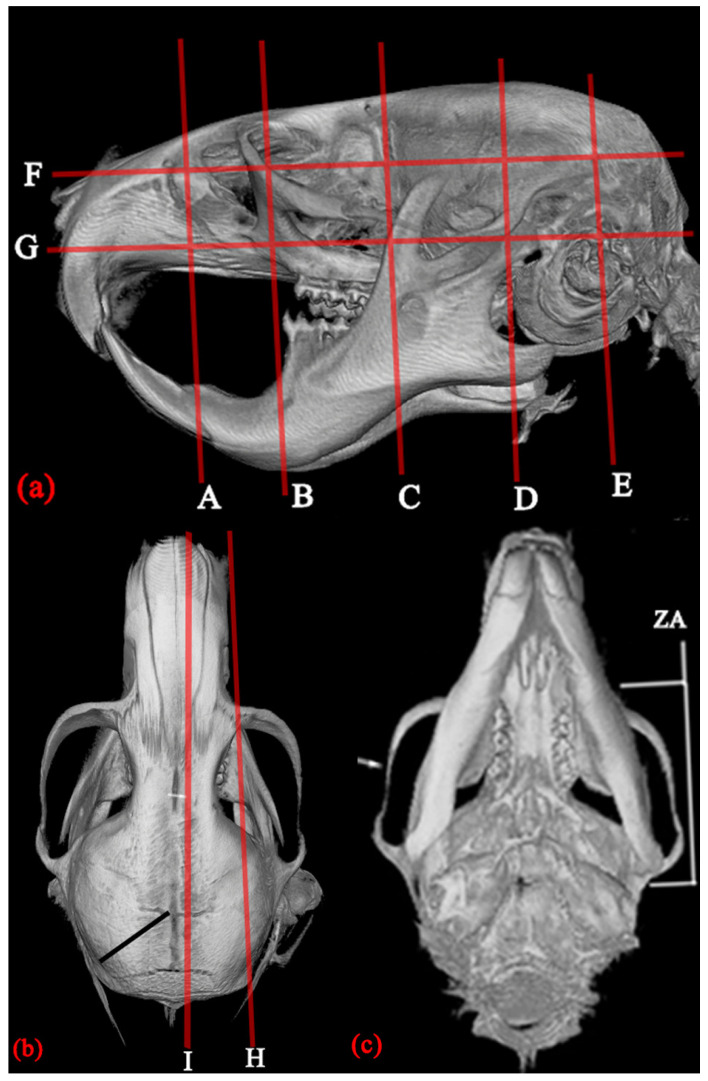
Volume rendered 3D-reformatted MCT images of the left lateral (**a**), dorsal (**b**), and ventral (**c**) views of a male Syrian hamster head. In (**a**,**b**), lines and large alphabetic letters indicate the approximate levels of the transverse (A–E), dorsal (F and G), and sagittal (I and H) gross, MCT, and MRI sectional planes included in Figure 4, Figure 5, Figure 6, Figure 7, Figure 8, Figure 9, Figure 10, Figure 11 and Figure 12. ZA: Zygomatic arch. White arrow: Zygomatic bone. Black line: Cranial suture.

Superficial head structures were demonstrated in the ventral and left lateral views to identify the major salivary glands and lymph nodes, as well as surface features of the muzzle, cheek pouch, and extraorbital lacrimal gland (Figure 1). Skull osteology was presented in the lateral, dorsal, and ventral aspects to evaluate the main external bony features (Figure 2 and Figure 3).

**Figure 4:**
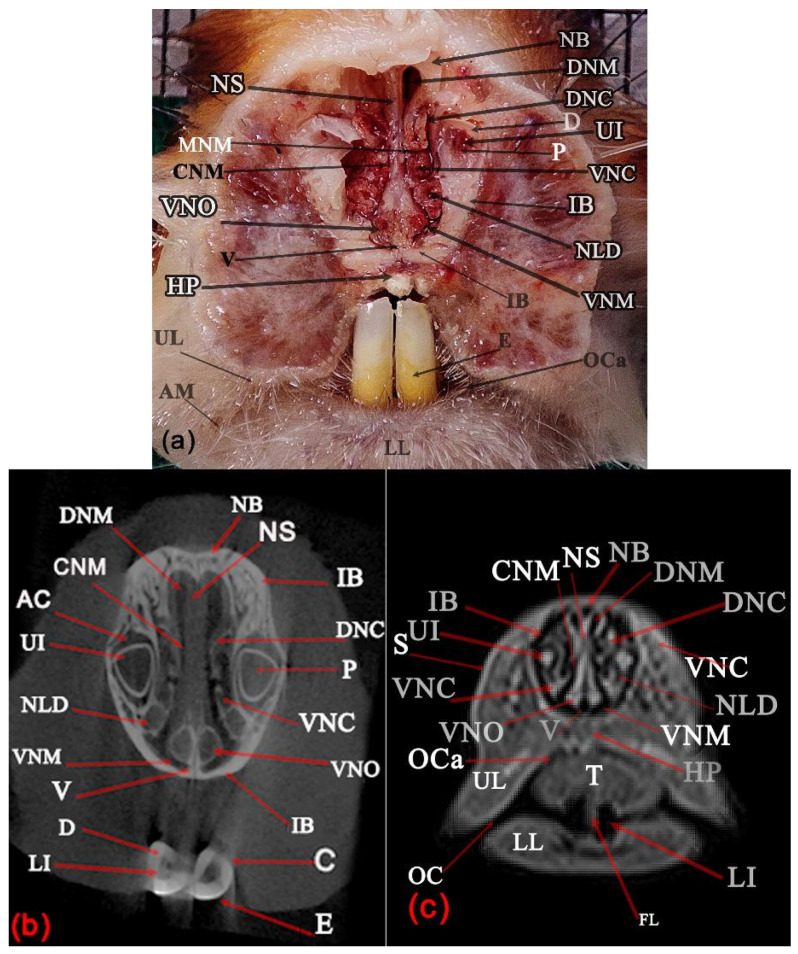
Transverse planes of a male Syrian hamster head: anatomical (**a**), MCT (**b**), and T1-weighted MR (**c**) images, corresponding to line A in (**a**). Rostral view: **AC:** Alveolar cavity of the tooth, **AM**: Angle of mouth, **C:** Cementum, **CNM**: Common nasal meatus, **D**: Dentin, **DNC**: Dorsal nasal concha, **DNM**, Dorsal nasal meatus, **E**: Enamel, **FL**: Frenulum linguae, **HP**: Hard palate, **IB**: Incisive bone, **LI**: Lower incisor, **LL**: Lower lip, **MNM**: Middle nasal meatus, **NB**: Nasal bone, **NLD**: Nasolacrimal duct, **NS**: Nasal septum, **OC**: Oral cleft, **OCa**: Oral cavity, **P**: Pulp cavity of maxillary incisor tooth, **S**: Skin, T: Tongue, **UI**: Upper incisor, **UL**: Upper lip, **V**: Vomer, **VNC**: Ventral nasal concha, **VNM**: Ventral nasal meatus, and **VNO**: Vomeronasal organ. Scale: 1 mm in (**a**).

**Figure 5:**
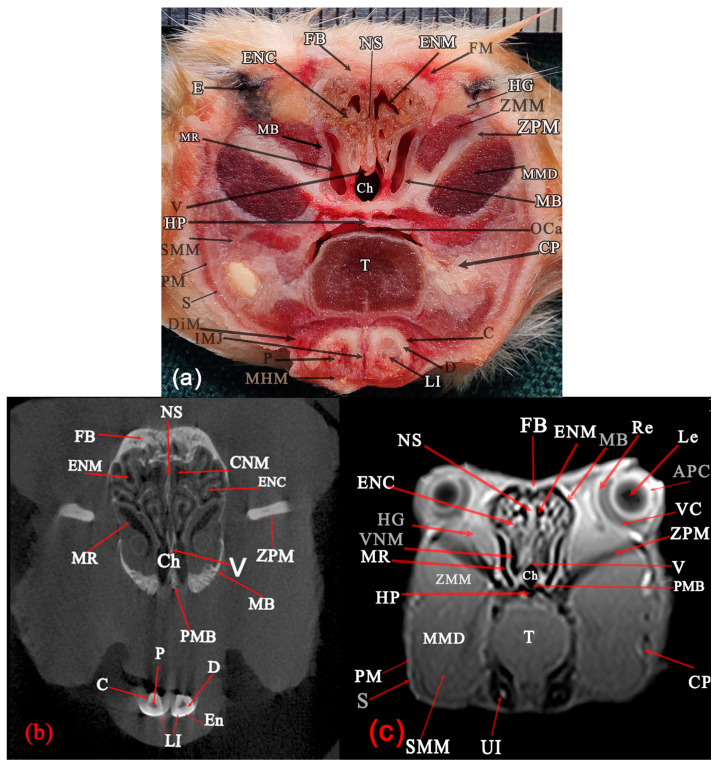
Transverse planes of a male Syrian hamster head: anatomical (**a**), MCT (**b**), and T1-weighted MR (**c**) images, corresponding to line B in (**a**). Rostral view: **APC**: Anterior and posterior chamber of eye, **C**: Cementum, **Ch**: Choanae, **CNM**: Common nasal meatus, **CP**: Cheek pouch, **D**: Dentin, **DiM**: Digastric muscle, **E**: Eye, **En**: Enamel, **ENC**: Ethmoidal nasal concha, **ENM**: Ethmoidal nasal meatus, **FB:** Frontal bone, **FM**: Frontal muscle, **HG**: Harderian gland, **HP**: Hard palate, **IMJ**: Intermandibular joint, **Le**: Lens, **LI**: Lower incisor, **MB**: Maxilla, **MHM**: Mylohyoid muscle, **MMD:** Deep part of masseter muscle, **MR**: Maxillary recess, **NS**: Nasal septum, **OCa**: Oral cavity, **P**: Pulp cavity of tooth, **PM**: Platysma muscle, **PMB:** Palatine process of maxilla, **Re:** Retina, **S**: Skin, **SMM**: Superficial part of masseter muscle, **T:** Tongue, UI: Upper incisor, **V**: Vomer, **VC**: Vitreous chamber, **VNM**: Ventral nasal meatus, **ZPM**: Zygomatic process of maxilla, and **ZMM**: Zygomaticomandibularis muscle. Scale: 1 mm in (**a**).

**Figure 6:**
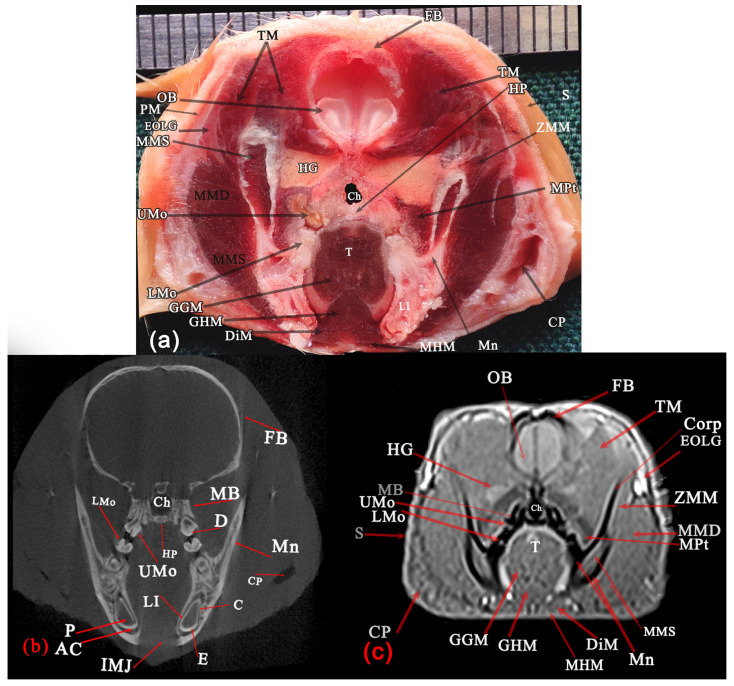
Transverse planes of a male Syrian hamster head: anatomical (**a**), MCT (**b**), and T1-weighted MR (**c**) images, corresponding to line C in (**a**). Rostral view: **AC**: Alveolar cavity of teeth, **C**: Cementum, **Ch**: Choanae, Corp: Coronoid process of mandible, **CP**: Cheek pouch, **D**: Dentin, **DiM**: Digastric muscle, **E**: Enamel, **EOLG**: Extraorbital lacrimal gland, **FB**: Frontal bone, **GGM**: Genioglossus muscle, **GHM**: Geniohyoid muscle, **HG**: Harderian gland, **HP**: Hard palate, **IMJ**: Intermandibular joint, **LI**: Lower incisor, **LMo**: Lower molar tooth, **MB**: Maxillary bone, **MHM**: Mylohyoid muscle, **MMD**: Deep part of masseter muscle, **MMS**: Superficial part of masseter muscle, **MPt**: Medial pterygoid muscle, **Mn**: Ramous of Mandible, **OB**: Olfactory bulb, **P**: Pulp of tooth, **PM**: Platysma muscle, **S**: Skin, **TM**: Temporal muscle, **T**: Tongue, **UMo**: Upper molar tooth, and **ZMM:** Zygomaticomandibularis muscle. Scale: 1 mm in (**a**).

**Figure 7:**
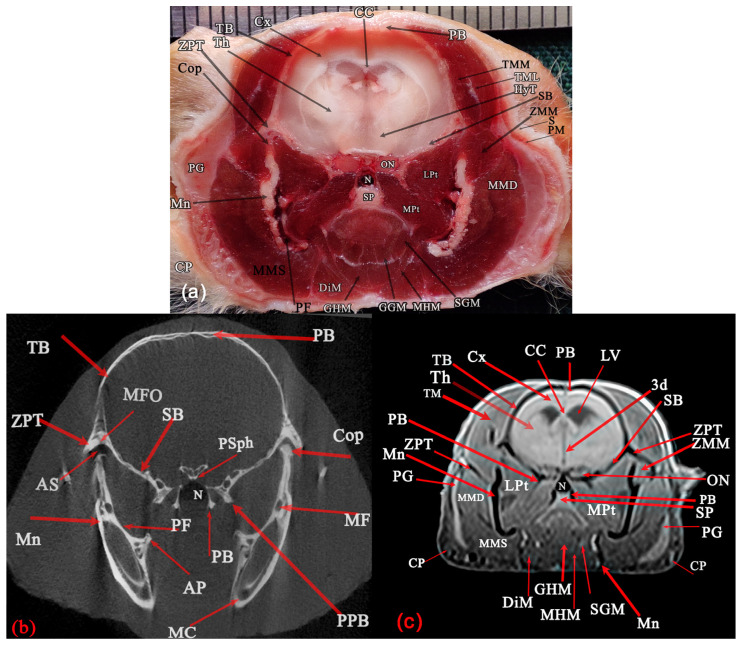
Transverse planes of a male Syrian hamster head: anatomical (**a**), MCT (**b**), and T1-weighted MR (**c**) images, corresponding to line D in (**a**). Rostral view: **3d**: Third ventricle, AP: Angular process, **AS**: Articular space of temporomandibular joint, **CC**: Corpus callosum, **Cop**: Condyloid process, **CP**: Cheek pouch, **Cx**: Cerebral cortex, **DiM**: Digastric muscle, **GGM**: Genioglossus muscle, **GHM**: Geniohyoid muscle, **HyT**: Hypothalamus, **LPt:** Lateral pterygoid muscle, **LV**: Lateral ventricle, **MC**: Mandibular canal, MF: Mandibular foramen, MFO: Mandibular fossa, **MHM**: Mylohyoid muscle, **Mn**: Ramus of Mandibular bone, **MMD**: Deep part of the masseter muscle, **MMS**: Superficial part of masseter muscle, **MPt**: Medial pterygoid muscle, **N**: Nasopharynx, **ON**: Optic nerve, **PB**: Pterygoid bone, **PF**: Pterygoid fossa, **PG**: Parotid salivary gland, **PM**: Platysma, **PPB:** Perpendicular process of palatine bone, **PSph**: Presphenoid bone, **S**: Skin, **SB**: Wing of presphenoid bone, **SGM**: Styloglossus muscle, **SP**: Soft palate, **TB**: Temporal bone, **Th**: Thalamus, **TM**: Temporal muscle, **TML**: Lateral part of the temporal muscle, **TMM**: Medial part of temporal muscle, **ZMM**: Zygomaticomandibular muscle, and **ZPT**: Zygomatic process of temporal bone. Scale: 1 mm in (**a**).

**Figure 8:**
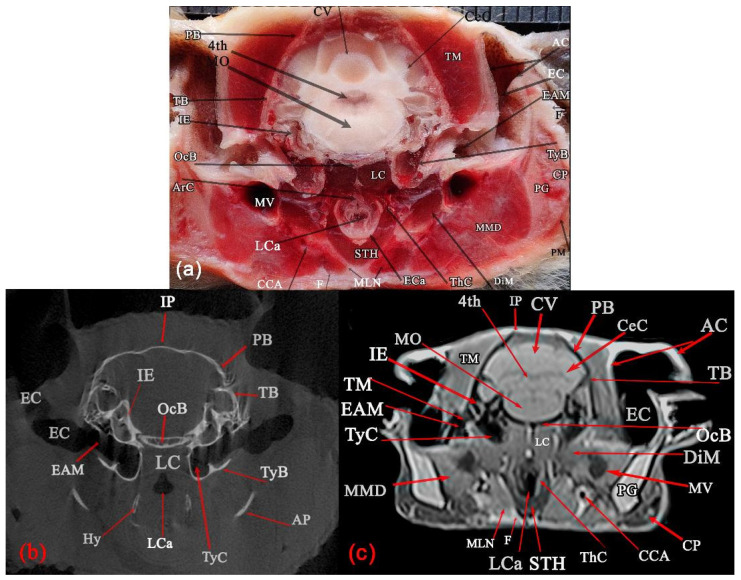
Transverse planes of a male Syrian hamster head: anatomical (**a**), MCT (**b**), and T1-weighted MR (**c**) images, corresponding to line E in (**a**). Rostral view: **4th**: Forth ventricle, **AC**: Auricular cartilage, **AP**: Angular process, **ArC**: Arytenoid cartilage, **CCA**: Common carotid artery, **Cec**: Cerebellum hemisphere, **CP**: Cheek pouch, **CV**: Vermis of cerebellum, **DiM**: Digastric muscle, **EAM**: External acoustic meatus, **EC**: Ear canal, **ECa**: Epiglottic cartilage, **F**: Fat tissue, **Hy**: Thyrohyoid bone, **IE**: Inner ear, **LC**: Longus capitis muscle, **LCa**: Laryngeal cavity, **IP**: Interparietal bone, **MLN**: Mandibular lymph node, **MMD**: Deep part of the masseter muscle, **MO**: Medulla oblongata, **MV**: Maxillary vein, **OcB**: Occipital bone, **PB**: Parietal bone, **PG**: Parotid salivary gland, **PM**: Platysma, **STH**: Sternothyrohyoid muscle, **TB**: Temporal bone, **ThC**: Thyroid cartilage, **TM**: Temporal muscle, **TyB**: Tympanic bulla, and **TyC**: Tympanic cavity. Scale: 1 mm in (**a**).

**Figure 9:**
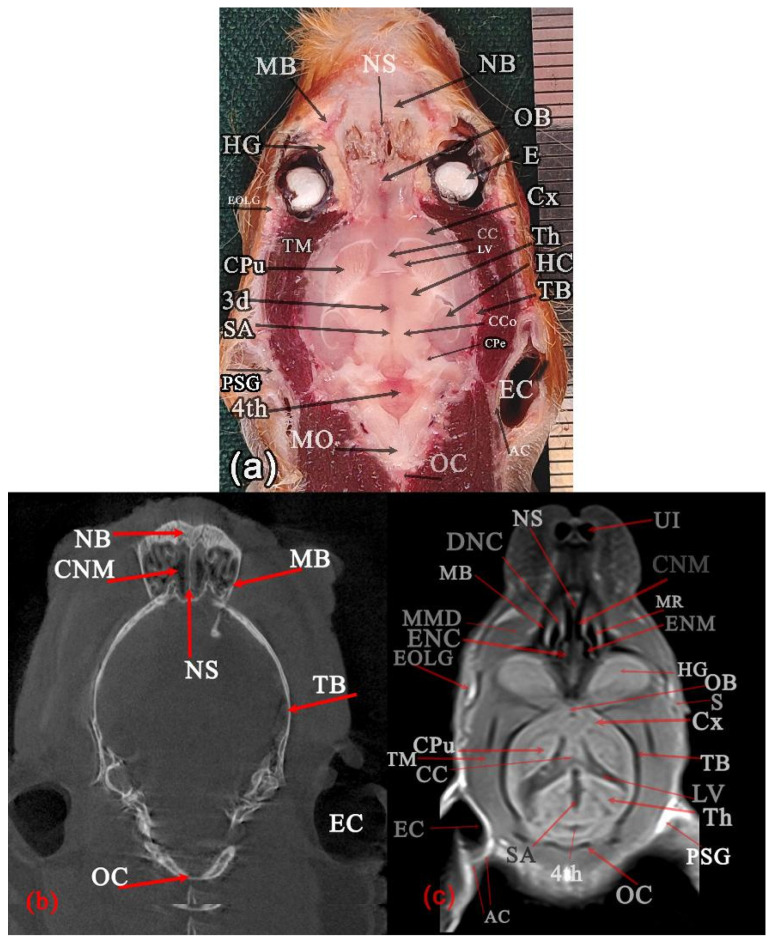
Dorsal planes of a male Syrian hamster head: anatomical (**a**), MCT (**b**), and T1-weighted MR (**c**) images, corresponding to line F in (**a**). Ventral segment: **3d**: Third ventricle, **4th**: Fourth ventricle, **AC**: Auricular cartilage, **CC**: Corpus callosum, **CCo**: Caudal colliculus, **CNM**: Common nasal meatus, **CPu**: Caudoputamen, **CP**e: Cerebral peduncle, **Cx**: Cerebral cortex, **DNC:** Dorsal nasal concha, E: Eye, **EC**: Ear canal, **ENC**: Ethmoidal nasal concha, **ENM**: Ethmoidal nasal meatus, **EOLG**: Extraorbital lacrimal gland, **HC**: Hippocampus, **HG**: Harderian gland, **LV**: Lateral ventricle, **MB**: Maxillary bone, **MMD**: Deep part of the masseter muscle, **MO**: Medulla oblongata, **MR:** Maxillary recess, **NB**: Nasal bone, **NS**: Nasal septum, **OB**: Olfactory bulb, **OC**: Occipital bone, **PSG**: Parotid salivary gland, **S**: Skin, **SA**: Sylvius aqueduct, **TB**: Temporal bone, **Th**: Thalamus, **TM**: Temporal muscle, and **UI**: Upper incisor teeth. Scale: 1 mm in (**a**).

**Figure 10:**
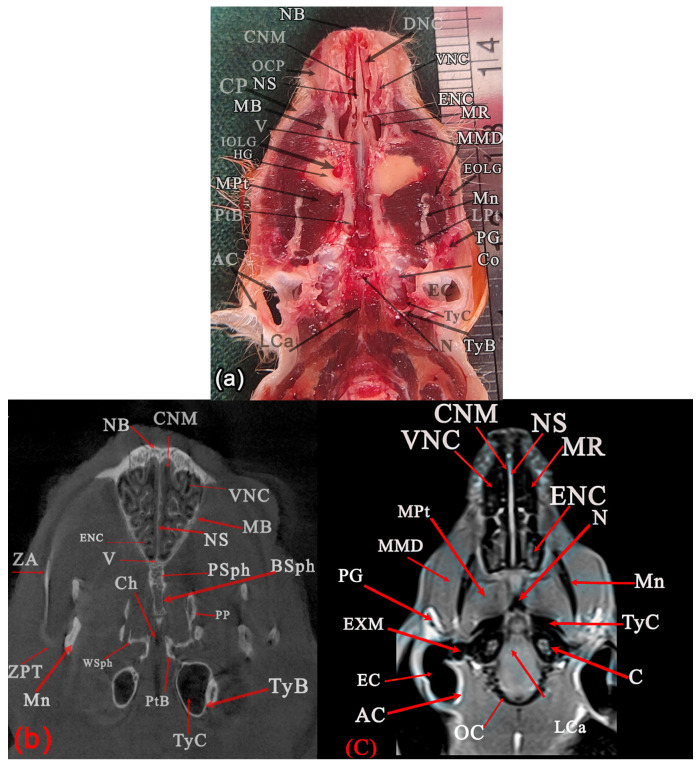
Dorsal planes of a male Syrian hamster head: anatomical (**a**), MCT (**b**), and T1-weighted MR (**c**) images, corresponding to line G in (**a**). Ventral segment: **AC**: Auricular cartilage, **BSph**: Body of Basisphenoid bone, **C**: Cochlea, **Ch**: Choana, **CNM**: Common nasal meatus, **Co:** Cochlea, **CP**: Check pouch, **DNC:** Dorsal nasal concha, **EC**: Ear canal, **ENC**: Ethmoidal nasal concha, **EXM**: External acoustic meatus, **EOLG**: Extraorbital lacrimal gland, **HG**: Harderian gland, **IOLG**: Intraorbital lacrimal gland, **LCa**: Longus capitis muscle, **LPt**: Lateral pterygoid muscle, **MB**: Maxillary bone, **MMD**: Deep part of the masseter muscle, **Mn**: Ramus of Mandible, **MPt**: Medial pterygoid muscle, **MR**: Maxillary recess, **N**: Nasopharynx, **NB**: Nasal bone, **NS**: Nasal septum, **OC**: Occipital bone, **OCP**: Orifice of the cheek pouch, **PG**: Parotid salivary gland, **PP**: Perpendicular palate of palatine bone, **PSph**: Body of Presphenoid bone, **PtB**: Pterygoid bone, **TyB**: Tympanic bulla, **TyC**: Tympanic cavity, **V**: Vomer bone, **VNC**: Ventral nasal conchae, **WSph**: Wings of sphenoid bone, **ZA**: Zygomatic arch, and **ZPT**: Zygomatic process of temporal bone. Scale: 1 mm in (**a**).

**Figure 11:**
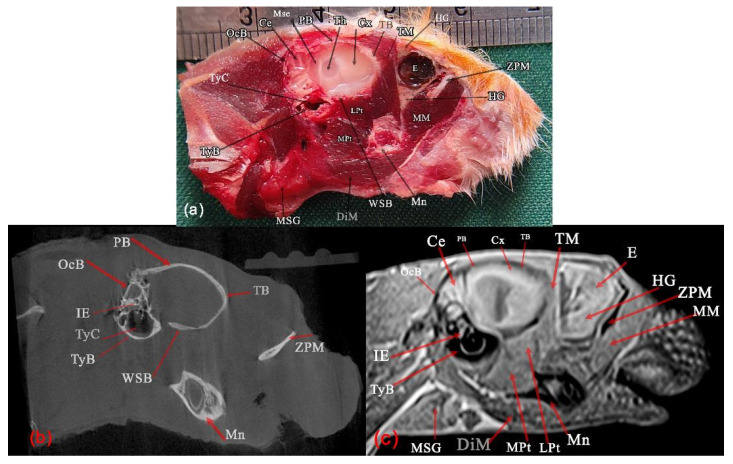
Paramedian planes of a female Syrian hamster head: anatomical (**a**), MCT (**b**), and T1-weighted MR (**c**) images, corresponding to line H in (**b**). Left medial view: **Ce**: Cerebellum, **Cx**: Cerebral cortex, **DiM**: Digastric muscle, **E**: Eye, **HG**: Hypophysial gland, **IE**: Inner ear, **LPt**: Lateral pterygoid muscle, **MM**: Deep part of the masseter muscle, **Mn**: Mandible, **MPt**: Medial pterygoid muscle, **Mse**: Mesencephalon, **MSG**: Mandibular salivary gland, **OcB**: Occipital Bone, **PB**: Parietal bone, **Th**: Thalamus, **TM**: Temporalis muscle, **TB**: Temporal bone, **TyC**: Tympanic cavity, **TyB:** Tympanic bulla, **WSB**: Wing of presphenoid Bone, **ZPM**: Zygomatic process of maxilla. Scale: 1 mm in (**a**).

**Figure 12:**
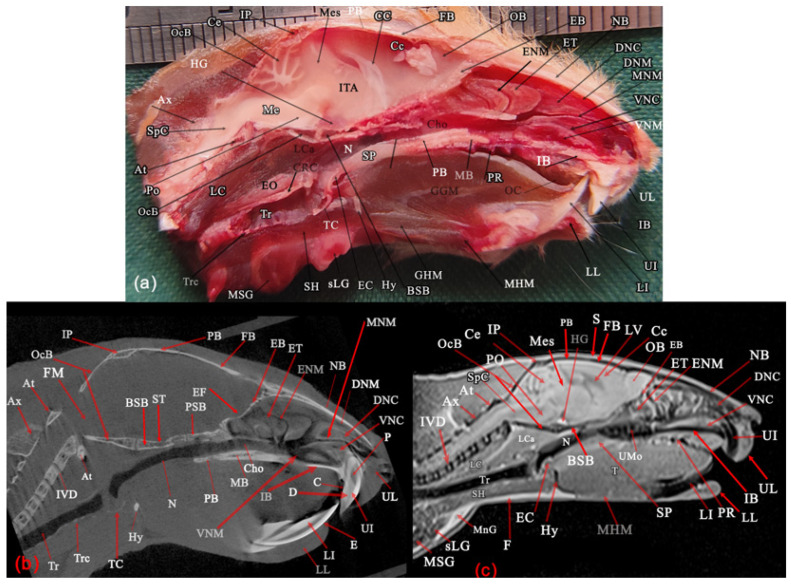
Median planes of a female Syrian hamster head: anatomical (**a**), MCT (**b**), and T1-weighted MR (**c**) images, corresponding to line I in (**b**). Left medial view: **At**: Atlas, **Ax**: Axis, **BSB**: Body of Basisphenoid Bone, **C**: Cementum, **CC**: Corpus callosum, **Ce**: Vermis of cerebellum, **Cc**: Cerebral cortex, **Cho**: Choanae, **CRC**: Cricoid cartilage, **D**: Dentine, **DNC**: Dorsal nasal concha, **DNM**: Dorsal nasal meatus, **EC**: Epiglottic cartilage, **EB**: Cribriform palate of Ethmoidal bone, **EF**: Ethmoid Fossa, **E**: Enamel, **ENM**: Ethmoidal nasal meatus, **EO:** Esophagus, **ET**: Ethmoidal nasal concha, **F**: Fat tissue, **FB**: Frontal Bone, **FM**: Foramen magnum, **GHM:** Geniohyoid muscle, **GGM**: Genioglossus muscle, **HG**: Hypophysial gland, **Hy**: Basihyoid bone, **IB**: Incisive bone, **IP**: Interparietal bone, **ITA**: Interthalamic adhesion, **IVD**: Intervertebral disk, **LC**: Longus colli muscle, **LCa**: Longus capitis muscle, **LI**: Lower Incisor, **LL**: Lower Lip, **LV:** Lateral ventricle, **MB:** Maxilla, **Me:** Medulla oblongata, **Mes**: Mesencephalon, **MHM**: Mylohyoid muscle, **MnG:** Mandibular lymph node, **MNM**: Middle nasal meatus, **MSG**: Mandibular salivary gland, **N**: Nasopharynx, **NB**: Nasal bone, **OB**: Olfactory bulb, **OC:** Oral cavity, **OcB**: Occipital bone, **P**: Pulp of tooth, **PB**: Parietal Bone, **Po**: Pons, **PR**: Palatine ridge, **PSB**: Body of Presphenoid bone, **S:** skin, **SH**: Sternohyoid muscle, **slG**: sublingual salivary gland, **SP**: Soft palate, **Sp**C: Spinal cord, **ST**: Sella Turcica, **T**: Tongue, **TC**: Thyroid cartilage, **Tr**: Trachea, **Trc**: Trachea cartilage, **UI**: Upper incisor tooth, **UL**: Upper lip, **UMo**: Upper molar teeth, **VNC**: Ventral nasal concha, and **VNM**: Ventral nasal meatus. Scale: 1 mm in (**a**).

The approximate levels of the dorsal, sagittal, and transverse sectional planes at which images were obtained were illustrated in the lateral (a) and dorsal (b) views of the 3D-reformatted MCT images (Figure 3). A total of twelve sectional planes were presented, including five serial transverse sections (Figure 4, Figure 5, Figure 6, Figure 7 and Figure 8), two dorsal sections (Figure 9 and Figure 10), and two sagittal sections (Figure 11 and Figure 12). The following anatomical landmarks were used to define the reference lines for these sectional planes: the level of the maxillary incisor teeth (Figure 4); the caudal part of the diastema, just rostral to the first molar (Figure 5); a level adjacent to the caudal margin of the orbital cavity (Figure 6); the level of the temporomandibular joint (Figure 7); the level of the external acoustic meatus (Figure 8); a level just dorsal to the occipital condyle (Figure 9); the level of the external acoustic meatus (Figure 10); the level of the orbital cavity (Figure 11); and the midline (Figure 12). Each figure was composed of a gross anatomical slice (a), an MCT image (b), and an MR image (c). All transverse sections were viewed from the rostral aspect. All clinically relevant structures of the Syrian hamster head were identified on these sections. T1-weighted images were found to provide superior clarity and tissue differentiation compared with T2-weighted images and were therefore considered the optimal modality for analysis in this study. In our specimens, the cerebrospinal fluid (CSF) within the ventricular system and subarachnoid space, as well as the parotid and mandibular salivary glands, the extraorbital lacrimal gland, and the harderian gland, were more clearly distinguishable from the surrounding structures on T1-weighted images.

### 3.1. Gross Anatomy

#### 3.1.1. Superficial Structures of the Head and Cranial End of the Neck

##### Cheek Pouch

The cheek pouches were described as paired, thin-walled, and distensible evaginations of the oral cavity, forming double-walled epithelial sacs. They were extended dorsocaudally along the lateral mandible from the mouth to the shoulder region, as well as from the ventral region to the ears, and were positioned between the skin and masticatory muscles (Figure 2a,b). The pouches were located over the parotid salivary gland, masseter muscle, and extraorbital lacrimal gland, but were not visible externally. The medial surface of each pouch was not covered by skin. The retractor sacculi was attached to the caudal pouch and was passed under its dorsomedial surface over the masseter to be inserted at the angle of the mouth (Figure 2a,b).

##### Major Salivary Glands

The major salivary glands identified in the Syrian hamster included the parotid, mandibular, and sublingual glands. Adjacent to the glands, lobulated brown fat structures were observed, which could be mistaken for lymph nodes or salivary glands (Figure 1a,c). The flat parotid glands were lobulated and extended from the ventral aspect of the ear base to the caudal margin of the mandibular angle, lying over the masseter muscle. Laterally, the gland was covered by the cheek pouch and surrounded by adipose tissue (Figure 1a,c). The parotid duct emerged from the medial surface of the caudal third of the gland and, together with the ventral buccal branch of the facial nerve, coursed laterally across the masseter (Figure 1a,c).

Upon removal of the skin, the mandibular salivary gland was observed in the ventral neck region. It exhibited a distinctly lobulated structure, partially covering the lateral neck and extending caudally toward the thoracic inlet. The gland was in rostrolateral contact with the mandibular lymph node, and the mandibular glands of both sides were in contact along the midline (Figure 1a,c).

The small sublingual gland was closely attached to the cranial–medial portion of the mandibular gland. It exhibited lighter coloration compared to the mandibular gland and had a uniform, non-lobulated structure. Laterally, it was situated adjacent to the mandibular lymph node (Figure 1a,c).

##### Extraorbital Lacrimal Glands

The oval, pinkish extraorbital lacrimal glands were subcutaneous and under the cheek pouch and over the deep part of the masseter and temporalis muscles. The gland was located caudoventral to the eye and rostrodorsal to the parotid salivary gland (Figure 1a).

##### Skull and Teeth

The skull was pyramidal in general form, with the face blending smoothly into the cranium without a dorsal “stop.” The skull was characterized by large orbits, large temporal fossae, incomplete postorbital bars, absence of an external sagittal crest, well-developed incisive bone, and prominent tympanic bullae. The dorsal cranial surface was almost flat, facial length was similar to cranial length, and cranial height was greater than facial height (Figure 1 and Figure 3). The zygomatic bone was a fine spicule positioned between the zygomatic processes of the maxilla and temporal bones to form the zygomatic arch, but was not involved in forming the orbital cavity or face. It was not always ossified in the examined animals. In macerated skulls, the zygomatic bone was absent, but it was visible in reconstructed 3D micro-CT images. Zygomatic arches were laterally curved and almost dorsally straight, with the long temporal process of the zygomatic bone joining the short zygomatic process of the temporal bone (Figure 1 and Figure 3). The incisive bone was well developed, forming the lateral face, with the infraorbital foramen located rostroventrally to the orbit (Figure 1 and Figure 3). The ventral border of the mandibular body was convex, containing a deep concavity, and the angular process projected caudally. The articular surface of the condyloid process was oriented rostrocaudally, and a prominent three-sided masseteric fossa was noted (Figure 1 and Figure 3). The dental formula was 2 (I 1/1, C 0/0, P 0/0, M 3/3) = 16, with incisors and molars separated by a relatively large diastema (Figure 1). The maxillary diastema was larger than the mandibular diastema. Large maxillary incisors were strongly curved, while smaller mandibular incisors were mildly curved. Tooth size decreased from rostral to caudal, with the first mandibular and maxillary molars being the largest (Figure 1 and Figure 3).

#### 3.1.2. Anatomical, MCT, and MRI Sections

Bony structures of the head were identified, including the nasal bone (Figure 4a–c, Figure 9a,b, Figure 10a,b and Figure 12a–c), vomer (Figure 4a–c, Figure 5a–c and Figure 10a–c), nasal septum (Figure 4a–c, Figure 5a–c, Figure 9a–c and Figure 10a–c), incisive bone (Figure 4a–c and Figure 12a–c), frontal bone (Figure 5a–c and Figure 12a–c), intermandibular joint (Figure 5a,c and Figure 6b), palatine process of maxilla (Figure 5b,c), zygomatic process of maxilla (Figure 5a–c and Figure 11a–c), mandible (Figure 11a–c), maxilla (Figure 5a–c, Figure 6a–c, Figure 9a–c, Figure 10a–c and Figure 12a–c), ramus of mandible (Figure 5a–c, Figure 7a–c and Figure 10a–c), condylar process of mandible (Figure 7a,b), presphenoid bone (Figure 7a and Figure 10b), articular space of temporomandibular joint, mandibular canal, and pterygoid fossa (Figure 7b), pterygoid bone (Figure 7b,c and Figure 10a,b), perpendicular palate of palatine bone (Figure 7b and Figure 10b), zygomatic arch (Figure 10b), zygomatic process of temporal bone (Figure 7a–c and Figure 10b), angular process, and thyrohyoid bone (Figure 8a), interparietal bone (Figure 8a–c and Figure 12a–c), external acoustic meatus (Figure 8a–c and Figure 10c), tympanic cavity, and tympanic bulla (Figure 8a–c, Figure 10a–c and Figure 11a–c), temporal bone (Figure 8a–c, Figure 9a–c and Figure 11a–c), parietal bone (Figure 8a–c, Figure 11a–c and Figure 12a–c), occipital bone (Figure 8, Figure 9, Figure 11a–c and Figure 12a–c), wing of presphenoid bone (Figure 10b and Figure 11a,b), body of basisphenoid bone (Figure 10b and Figure 12a–c), body of presphenoid bone (Figure 10b and Figure 12b), ethmoid fossa, sella turcica, and foramen magnum (Figure 12b), intervertebral disk (Figure 12b,c), cribriform palate of ethmoidal bone, atlas, axis, and basihyoid bone (Figure 12a–c).

In micro-CT images, bony structures appeared radiopaque (bright), while air-filled regions were hypodense (dark or black) due to low X-ray attenuation. In micro-CT images, the articular space of the temporomandibular joint appeared as a hypodense gap between bones, while the articular disk was not visualized. The auditory ossicles were not delineated. In micro-CT images, the intermandibular joint (mandibular symphysis) appeared cartilaginous as a narrow radiolucent gap between the mandibular halves. In micro-CT images, the cheek pouches were identifiable laterally as air-filled, radiolucent cavities. On T1-weighted MRI, bony and air-filled structures generally appeared hypointense (dark). Meanwhile, some parts of the bones located within the nasal cavity, including the nasal septum, were easy to assess because of the distinct mucosal covering.

Tooth structures were identified including alveolar cavity of the tooth (Figure 4b and Figure 6b), cementum, dentin, enamel, pulp cavity of tooth (Figure 4a,b, Figure 5b, Figure 6b and Figure 12b), upper incisor (Figure 4a–c, Figure 8c and Figure 12a–c), lower incisor (Figure 4b,c, Figure 5a–c, Figure 6a,b and Figure 12a–c), lower molar tooth (Figure 6a–c), and upper molar (Figure 6a–c and Figure 12c).

On the MCT images, dental tissues showed distinct patterns: enamel was the brightest, dentin light gray, and cementum a thin, slightly less dense layer, often hard to distinguish from dentin. The pulp cavity appeared darkest in micro-CT due to the lack of mineral content, but hyperintense on T1-weighted images due to high water content. On T1-weighted MRI, tooth structures generally appeared hypointense (dark). Differentiation of enamel, dentin, and cementum on T1-weighted images was not possible.

The muscular and integumentary of systems of the head were identified, including the longus capitis muscle (Figure 8a,c, Figure 10a,c and Figure 12a,c), sternohyoid muscle (Figure 12a,c), digastric muscle (Figure 5a,c, Figure 6a,c, Figure 7a,c, Figure 8a,c and Figure 11a,c), medial pterygoid muscle (Figure 6a,c, Figure 7a,c, Figure 10a,c and Figure 11a,c), lateral pterygoid muscle (Figure 7a,c, Figure 10a,c and Figure 11a,c), longus colli muscle (Figure 12a,c), skin (Figure 4c, Figure 5a,c, Figure 6a,c, Figure 7a,c and Figure 9c), superficial part of masseter muscle (Figure 6a,c and Figure 7a,c), zygomaticomandibularis muscle (Figure 5a,c, Figure 6a,c and Figure 7a,c), deep part of the masseter muscle (Figure 5a,c, Figure 6a,c, Figure 7a,c, Figure 8a,c, Figure 9c, Figure 10a,c and Figure 11a,c), sternothyrohyoid muscle (Figure 8a,c and Figure 12a,c), platysma (Figure 7a and Figure 8a), temporal muscle (Figure 6c, Figure 7c, Figure 8a,c, Figure 9a,c and Figure 11a,c), genioglossus muscle (Figure 6a,c and Figure 7a,c), lateral and medial parts of the temporal muscle (Figure 6a and Figure 7a), geniohyoid muscle (Figure 6a,c, Figure 7a,c and Figure 12a), styloglossus muscle (Figure 7a,c), mylohyoid muscle (Figure 7a,c and Figure 12a,c), and fat tissue (Figure 8a,c and Figure 12c). Muscles and fats were poorly visible or radiolucent in non-contrast micro-CT and typically indistinguishable without contrast enhancement. On T1-weighted MRI, head muscles showed intermediate signal intensity—brighter than bone but darker than fat. Fat appeared hyperintense, and skin was slightly brighter than muscle.

Nervous structures of the head were identified, including the olfactory bulb (Figure 6a,c, Figure 9a,c and Figure 12a,c), cerebral cortex (Figure 6, Figure 7a,c, Figure 9a,c, Figure 11a,c and Figure 12a,c), hypothalamus (Figure 7a), thalamus, (Figure 7a,c, Figure 9a,c and Figure 11a), lateral ventricle (Figure 7a,c and Figure 9c), optic nerve (Figure 7a,c), Third ventricle (Figure 7a,c and Figure 9a,c), forth ventricle (Figure 8a,c and Figure 9a,c), cerebellum hemisphere (Figure 8a,c and Figure 11a,c), vermis of cerebellum (Figure 8a,c and Figure 12a,c), hypophysial gland (Figure 12a,c), mesencephalon (Figure 11a and Figure 12a,c), thalamus, (Figure 9a,c and Figure 11a), interthalamic adhesion (Figure 12a,c), cerebral hemisphere (Figure 12a,c), corpus callosum (Figure 7a,c, Figure 9a,c and Figure 12a,c), medulla oblongata (Figure 8a,c, Figure 9a and Figure 12a,c), spinal cord (Figure 12a,c), sylvius aqueduct (Figure 9a,c), caudal colliculus (Figure 9a), caudoputamen (Figure 9a,c), cerebral peduncle (Figure 9a), and hippocampus (Figure 9a).

Nervous tissues were poorly visible or radiolucent in non-contrast micro-CT and typically indistinguishable without contrast enhancement. The contrast between gray and white matter was low in the T1-weighted MRI and showed similar signal intensities. In the transverse T1-weighted image, the cerebellum (mostly gray matter) had intermediate signal intensity and was darker than the medulla oblongata (mostly white matter), which had high signal intensity (Figure 8c). On the T1-weighted MRI, cerebrospinal fluid in the brain ventricles and subarachnoid space also appeared black, reflecting its very low signal.

Respiratory structures of the head were identified, including the choanae (Figure 5a–c, Figure 6a–c, Figure 10b and Figure 12a,b), dorsal nasal concha, and dorsal nasal meatus (Figure 4a–c, Figure 9b,c and Figure 12a–c), middle nasal meatus (Figure 4c and Figure 12a,b), ventral nasal meatus (Figure 4a–c, Figure 5c and Figure 12a,b), ventral nasal concha (Figure 4a–c, Figure 10a–c and Figure 12a–c), common nasal meatus (Figure 4a–c, Figure 5b, Figure 9b,c and Figure 10a–c), ethmoidal nasal concha, ethmoidal nasal meatus (Figure 5a–c, Figure 9a,c, Figure 10a,c and Figure 12a–c), maxillary recess (Figure 5a–c, Figure 9c and Figure 10a,c), nasopharynx, (Figure 7a–c, Figure 10a,c and Figure 12a–c), arytenoid cartilage, laryngeal cavity (Figure 8a,c), thyroid cartilage (Figure 8a,c and Figure 12a), trachea (Figure 12a–c), trachea cartilage (Figure 12a–c), cricoid cartilage (Figure 12a), epiglottic cartilage (Figure 8a and Figure 12a,c), nasolacrimal duct (Figure 4a–c), and vomeronasal organ (Figure 4a–c).

In micro-CT images, the nasolacrimal duct was located ventromedially to the upper incisor’s reversed crown. In micro-CT images of the maxillary recess and choanae, meatuses appeared as air-filled, radiolucent cavities. In micro-CT images, the nasopharynx and nasal meatuses appeared black due to air content. In micro-CT images, the laryngeal cartilages showed intermediate radiodensity, clearly distinguishable from adjacent soft tissues but less than calcified structures. On the T1-weighted image, the conchae and turbinates, as well as the lumen of the nasal cavity, laryngeal cavity, glottis, and trachea, were easily assessed because of the distinct mucosal covering. On the T1-weighted image, the vomeronasal organ appears as an oval or tubular structure with intermediate to slightly hyperintense signal intensity.

Digestive structures of the head were identified, including the angle of mouth (Figure 4a), frenulum linguae, and oral cleft (Figure 4a,c), tongue (Figure 4c, Figure 5a,c, Figure 6b,c and Figure 12b,c), lower lip (Figure 4a,c and Figure 12a–c), upper lip (Figure 12a–c), oral cavity (Figure 4a, Figure 5a and Figure 12a), cheek pouch (Figure 4a,c, Figure 5a–c, Figure 6a,b, Figure 7a,b and Figure 10c), mandibular lymph node (Figure 8a,c and Figure 12a,c), parotid salivary gland (Figure 7a,c, Figure 8a,c, Figure 9a,c and Figure 10a,c), soft palate (Figure 7a,c and Figure 12a), sublingual salivary gland (Figure 12a,c), mandibular salivary gland (Figure 11a,c and Figure 12a,c), palatine ridge, and esophagus (Figure 12a,c), and hard palate (Figure 4a,c and Figure 5a,c).

In micro-CT images, the cheek pouches were identifiable laterally as air-filled, radiolucent cavities. On T1-weighted MRI images, the cheek pouch showed inner mucosal folds and a lumen as small dark cavities and appeared slightly darker than the surrounding muscles, making it easily distinguishable. On T1-weighted MRI images, the parotid salivary gland appeared homogeneous with high signal intensity. On T1-weighted MRI images, the mandibular lymph node was homogeneous, hypointense relative to fat, and nearly isointense to adjacent muscles. On the T1-weighted MRI, the mandibular salivary gland appeared heterogeneous with low to moderate signal intensity. Due to its close relation with the small sublingual gland, its borders could not be clearly distinguished, and delineation was only possible with reference to anatomical sections. In the median plane, the individual borders of the mandibular salivary gland and its lymph node were difficult to delineate, and the gland area could only be identified with reference to the corresponding anatomical section. On T1-weighted images, the lumen appeared hypointense (dark), while air-filled structures, such as the oral cavity.

Eye structures were identified, including the anterior and posterior chamber of eye, retina, vitreous chamber, and lens (Figure 5c), harderian gland (Figure 5a,c, Figure 6a,c, Figure 9a,c, Figure 10a,c and Figure 11a,c), extraorbital lacrimal gland (Figure 6a,c, Figure 9a,c and Figure 10a), and eye (Figure 5a, Figure 9a and Figure 11a–c). Only in the gross section, a small round intraorbital lacrimal gland was red and located at the medial part of the orbit, adjacent to the harderian gland (Figure 10a).

No sex differences were observed in head anatomy and imaging, except for the harderian gland. In adult females, the harderian gland exhibited numerous dark spots in gross cross-sections; however, this feature was detectable only in anatomical sections (Figure 9, Figure 10 and Figure 11) and not in micro-CT or MRI images. On the gross sections, the lobulated harderian gland appeared as a light-yellow mass, occupying the ventromedial and posterior regions of the ocular bulb. On the T1-weighted MRI, the harderian gland was relatively hyperintense compared to muscle. On the T1-weighted MRI, the extraorbital lacrimal gland was homogeneous and exhibited high signal intensity. The relative signal intensity on T1-weighted images was as follows: lens capsule (cortex) > masticatory muscles > anterior/posterior chambers and vitreous > lens > bone, with air appearing black. The lens occupied a large proportion of the globe.

Ear structures were defined, including the ear canal (Figure 8a–c, Figure 9a–c and Figure 10a–c), auricular cartilage (Figure 8a,c, Figure 9b,c and Figure 10a–c), inner ear (Figure 8a–c and Figure 11b,c), and cochlea (Figure 10a,c). In the micro-CT image, the ear canal lumen was seen as hypodense (black) due to air content. On T1-weighted MRI images, auricular cartilage was bright and well-defined with intermediate to high signal.

The vascular system included the common carotid artery and maxillary vein (Figure 8a,c). They were signal void in the T1-weighted MRI images, while they were not visible in the MCT images.

## 4. Discussion

### 4.1. Technical Aspects

As seen in other rodents and rabbits, soft tissues, organs, and fluid-filled spaces were clearer on MRI than on CT or micro-CT (MCT). On the other hand, CT and MCT gave better images of bones and teeth. The air-filled spaces in the respiratory system (maxillary recess, nasal passages, laryngeal cavity, and trachea), digestive system (oral cavity), and sensory system (ear canal), as well as the tympanic bulla, appear black on both MCT/CT and MRI images [18,19,20,21,48]. Most of the important head structures could be clearly seen on micro-CT and MRI images, providing baseline information to help identify and interpret lesions.

The small size of SHs makes head anatomy difficult to visualize with conventional CT. Micro-CT (MCT), however, provides clear delineation of nearly all bony structures, air-filled cavities, and dental anatomy, demonstrating high diagnostic potential. Compared with helical CT, MCT offers greater bone sensitivity [48]. While consistent with our findings, De Rycke et al. [48] reported low soft tissue contrast in the rabbit head using MCT. While conventional CT cannot reliably detect small orthopedic lesions in rodents or rabbits, MCT is suitable for evaluating minor bone fractures in any anatomical region [49]. 

Veterinary MRI systems typically operate between 0.2 and 3 Tesla. Lower-field MRIs are more affordable and easier to use but produce lower-resolution images, require longer scan times, extended anesthesia, and are more prone to motion artifacts. In smaller species, including rabbits, 3 Tesla MRI offers clear advantages for detailed evaluation of the brain and surrounding bony structures [24]. Our findings confirm that 3 Tesla MRI enables clear visualization of clinically relevant head structures in SHs, supporting its value for enhancing diagnostic accuracy. Despite its usefulness, the high expense and limited accessibility of this equipment prevent its regular use in routine rodent veterinary care [20].

Due to the small size of SHs, a 3 Tesla MRI was performed using T1- and T2-weighted sequences in transverse, sagittal, and dorsal planes. T1-weighted images consistently provided superior resolution and clearer depiction of key head structures, consistent with findings in rabbits [19] and feline nasal and paranasal regions [12,13]. Fat appeared bright and fluid dark on T1-weighted images, enhancing tissue contrast and reducing noise and blurring. T2-weighted sequences were particularly effective for detecting fluid-related pathological changes [12,13].

One of the most commonly used slices is the transverse plane, which effectively and easily determines the dispositions and relations of the various head structures relative to each other. This plane is particularly suitable for diagnostic dental diseases [18]. For less experienced clinicians, the head sagittal plane is helpful for quickly understanding the topography of the head arrangements in the transverse plane. Sagittal images allow simultaneous evaluation of the rostral and caudal aspects of the head on a single image. However, sagittal images do not allow symmetric comparison between the left and right sides of the head, which would make clinical information less relevant. Moreover, using the dorsal plane can help identify the degree of filling or displacement of the head arrangements towards the right and left sides. Ultimately, employing three planes (transverse, sagittal, and dorsal) provides a more general view of the entire head, allowing for the easier characterization of disease processes.

Among the available literature on the morphology of rats, guinea pigs, Syrian hamsters, mice, porcupines, and rabbits, accessible sources for anatomical slices are reported in rabbits, porcupines, and guinea pigs [18,19,20,21,24]. Transverse and sagittal sections were made from frozen rabbit, porcupine, and guinea pig heads, with dorsal sections additionally prepared in rabbits. This study presents, for the first time, colorful frozen anatomical slices of the Syrian hamster head in all anatomical planes.

### 4.2. Imaging Anatomical Features

Due to the lack of investigations on the sectional anatomy and imaging of the head in common laboratory rodents, our findings were also compared with the available publications on CT/MRI with or without sectional anatomy of the rabbit head [18,19,48] and gross anatomical textbooks and atlases in common laboratory rodents. Consequently, important comparative morpho-topographical differences and similarities in head structures between the examined hamsters and the aforementioned species were revealed. Such comprehensive knowledge of the imaging anatomy in normal laboratory animals is essential for clinical diagnosis and therapeutic or surgical planning, particularly in hamsters, which commonly present with head-related disorders as well as appropriate selection of species as translational research models [1,4].

#### 4.2.1. Skull Bones

As with rabbit and porcupine heads [19,20], MRI was effective in visualizing the skull bones and teeth in SHs.

In rabbits [48,50], the maxilla is wider than the mandible. In SHs, the mandible is wider than the maxilla on MCT, similar to mice, rats, and cavies [50,51,52]. In SHs, the mandibular symphysis remains cartilaginous on MCT, as in mice, rats, cavies, and rabbits [18,48,51,53,54,55,56,57,58,59]. In rabbits, broad, interlocking symphyseal palates make the joint nearly immobile [58]. In rats and mice, the symphysis allows some mandibular movement, visible at the lower incisors [50]. The masseteric fossa is present in SHs in 3D MCT and dried skull observations, similar to that in rats, mice, and rabbits, but unlike cavies [18,48,51,53,54,55,56,57,58,59]. In SHs, the mandibular condyle is oriented rostrocaudally on 3D MCT and dried skulls, similar to mice and rats [51,53,54]. In rabbits, it is triangular, with the base rostral and apex caudal [18,48,56,57,58,59].

In SHs, the zygomatic bone lies between the zygomatic processes of the maxilla and temporal bones, forming a zygomatic arch on 3D MCT and dried skulls, as in rats, mice, cavies, and rabbits [18,48,51,53,54,55,56,57,58,59]. Like mice and rats [60,61], the zygomatic arch of SHs is elongated and thin on 3D MCT and dried skulls. In contrast, it is wide in cavies [55,56] and rabbits [18,48,56,57,58,59]. In rabbits, the zygomatic arch is nearly straight in dorsal and lateral views [18,48,56,57,58,59], whereas in SHs it is distinctly curved along its entire length, as shown by 3D MCT and dried skull evaluations, resembling the morphology seen in rats, mice, and cavies [18,51,53,54,55,56]. In SHs, the zygomatic arch shows a prominent zygomatic process of the maxilla on 3D MCT and dried skulls, as in mice, rats, and cavies [51,53,54,55,56].

The orbital cavity in SHs is nearly oval on 3D MCT and dried skulls, similar to rats and mice [51,53,54,55]. In cavies and rabbits, the orbit is roughly circular [18,48,56,57,58,59]. In SHs, the caudal wall of the orbit is absent on 3D MCT and dried skulls. In rabbits and cavies, however, the orbital wall is fully developed [18,48,55,56,57,58,59].

In rabbits, the external auditory meatus is broad and opens dorsolaterally [18,48,56,57,58,59]. In SHs, it is large and opens laterally, similar to mice and rats [51,53,54]. In cavies, it is small and laterally oriented [55,56].

In rabbits, the tympanic bullae are round and do not extend beyond the occipital bone [19,48,56,57,58,59]. In cavies, the bullae are large, oval, or trapezoid, contain tympanic cells, and remain within the occipital condyles [55,56]. In rats and other rodents, they are relatively small [53]. In SHs, the bullae extend ventrally beyond the occipital condyles and lack tympanic cells on 3D MCT and dried skulls, similar to rats and mice [51,53,54].

In rodents, the interparietal bone lies between the parietal and occipital bones, forming part of the dorsal calvaria. In mice and rats, it is well developed and visible externally [51,53,54]. In SHs, a triangular interparietal bone was seen on 3D MCT and dried skulls, similar to mice, rats, and rabbits [19,48,56,57,58,59]. It is absent in most adult cavies [55], though illustrated in anatomical atlases [56].

#### 4.2.2. Tooth Structures

Compared with CT scans of rabbit and porcupine heads [18,21], the mineralized dental tissues of Syrian hamsters (SHs)—including enamel, dentin, and cementum—were much more clearly visible on micro-CT (MCT) images. This finding is consistent with previous studies using MCT in rabbits [48]. Micro-CT is widely used to diagnose dental diseases in rabbits and other rodents [4], and these findings indicate it is a promising tool for detecting dental conditions in Syrian hamsters (SHs). Its high resolution allows early and precise identification of pathological changes that may be less apparent on helical CT [48].

All rabbit teeth are hypsodont [48,50]. In SHs, the incisors are hypsodont, while the cheek teeth are brachydont on MCT, similar to other rodents. Among small pet rodents, Cricetidae have the shortest brachydont cheek crowns [50,52,59]. SHs have 16 teeth on 3D MCT and dried skulls, similar to mice and rats [51,53,54,59]. Cavy-like rodents have 20–22 teeth, and rabbits have 28 [48,55,56,57,58,59]. Like other rodents and rabbits [48,51,53,54,55,56,57,58,59], SHs lack canine teeth on 3D or MCT, and dried skulls. SHs, like rats and mice [51,53,54,59], also lack premolars, whereas cavies and rabbits have them [48,55,56,57,58,59].

Consistent with other rodents [51,53,54,59], SHs have a single pair of maxillary incisors on 3D or MCT, as is also observable with dried skulls, whereas rabbits have one maxillary incisor per quadrant [48,56,57,58]. In rabbits, the maxillary incisors have enamel only on the labial side, while the mandibular incisors are enameled on both labial and lingual surfaces [48,50,52]; in cavy-like rodents, enamel is absent only on the lingual or palatal surfaces [50], and in rat-like rodents, enamel covers only the labial surface [50,51,52], consistent with transverse MCT observations in SHs. In SHs, the occlusal surfaces of the maxillary and mandibular incisors do not contact, as seen on 3D MCT and with dried skulls, a condition similar to mice [50,51,52] but unlike rabbits, cavies, and rats [48,50,52]. The labial surface of incisors is yellow–orange in cavy- and squirrel-like rodents, rabbits [50,52,59], and rat-like rodents [50,51,52], and SHs show a similar pattern on sectional anatomy.

#### 4.2.3. Jaw-Closing Muscles

The morphology of the jaw-closing muscles has long been used to classify rodents into subgroups [33,51,62,63], yet such data are lacking for Syrian hamsters (SHs). Across rodent species, including rat-like (mice and rats) and cavy-like (guinea pigs) groups, the masseter muscle typically comprises three layers—superficial, deep, and zygomaticomandibularis [33,51,62,63]—with the deep masseter showing considerable intergroup variation.

In SHs, the deep masseter originates on the rostrum, as demonstrated by MRI and sectional anatomy, similar to rats and mice [33,51,62,63], whereas in cavies, it is smaller and does not extend into this region [33]. The superficial masseter in SHs also extends rostrally onto the rostrum based on MRI and sectional observations, resembling the condition in rats, mice, and cavies [33,51,62,63]. The medial and lateral pterygoid muscles of SHs, observed via MRI and sectional anatomy, more closely resemble those of rats and mice than cavies, although these muscles generally show minimal variation among rats, cavies, and squirrel-like rodents [33,51,62,63]. The temporalis muscle of SHs, identified as the second-largest masticatory muscle on MRI and sectional anatomy, consists of distinct lateral and medial parts similar to mice and rats [33,51,62], whereas in cavies, it is relatively small and lacks this bipartite structure [33]. Collectively, these anatomical characteristics indicate that the jaw-closing muscle morphology in Syrian hamsters (SHs) aligns more closely with the pattern described for myomorph rodents.

#### 4.2.4. Paranasal Sinuses

Rodent sinus system anatomy differs markedly from that of lagomorphs, such as rabbits. In rodents, the sinus system shows both intra- and interspecific variation. Porcupines possess maxillary, frontal, sphenoid, and dorsal and ventral conchal sinuses on CT and sectional anatomy. Rabbits, in contrast, have paired dorsal conchal, maxillary, and frontal sinuses [18,48], with a sphenoid sinus also reported in veterinary texts [58]. Rats and mice exhibit a simpler pattern, with only a maxillary recess [51]. Syrian hamsters (SHs) display a sinus arrangement similar to other myomorph rodents on micro-CT and sectional anatomy. These findings suggest that the sinus system of myomorph rodents is poorly developed, and differences in sinus anatomy can be used to distinguish rodent groups from each other and from lagomorphs using CT imaging and sectional anatomical studies.

#### 4.2.5. Major Salivary Glands

No descriptions of the major salivary glands have been reported in MRI studies of rabbits or porcupines [19,20,22]. In contrast, the present study provides data on the signal intensity and homogeneity of the parotid and mandibular salivary glands in Syrian hamsters (SHs).

Rabbits and cavies possess four pairs of major salivary glands—parotid, mandibular, sublingual, and zygomatic [19,58]—whereas the zygomatic gland is absent in mice, rats [46,51,53,60], and SHs, as confirmed by MRI, sectional, and gross anatomical observations. In rabbits and cavies [19,46,55,58], the parotid gland is larger than the mandibular gland; however, in guinea pigs, mice, rats [46,51,53,55,60], and SHs, the mandibular gland is larger, a finding supported in the present study by gross anatomy and MRI. The mandibular gland shows species-specific positional variation: in rabbits, it is located at the mandibular angle [19,46,55], while in SHs, it occupies much of the ventral cervical region, similar to mice and rats [46,51,53,60]. Additionally, in SHs, the sublingual gland lies close to the rostral pole of the mandibular gland within the ventral cervical region, as confirmed by gross anatomy, anatomical sections, and MRI, resembling the arrangement reported in mice and rats [46,51,53,60]; this configuration has not been described in rabbits or cavies [19,46,58].

#### 4.2.6. Vomeronasal Organ

The vomeronasal capsule, a rigid protective structure of bone or cartilage, varies among species in serial histological transverse sections. Transverse MCT of the SH’s head reveals a predominantly ossified vomeronasal capsule, a feature also reported in certain rodents, such as rats and mice [64]. By contrast, in most mammals, including ungulates and carnivores, it is cartilaginous. In rabbits, the rostral and caudal portions of the capsule are cartilaginous, whereas the middle segment is bony [65]. Similarly, in some mole rat specimens, the capsule is either fully bony or mostly cartilaginous [61]. The architecture of the vomeronasal cartilage is taxon-specific. In Lagomorpha (rabbits), a unique double envelope of bone and cartilage is observed, similar only to young Rodentia, in which the cartilaginous capsule disappears a few weeks postnatally, leaving only the bony envelope [65].

The vomeronasal lumen shows intraspecific variation in serial histological transverse sections. In most mammals, including rabbits, it is typically crescent-shaped [65]; however, in rats, the rostral portion is round, whereas the caudal part may be either crescent [66] or round-shaped [64]. Similarly, micro-CT of rostral transverse sections in Syrian hamsters reveals an oval lumen, a morphology also observed in some mole rat species [61].

It should be noted that variations in vomeronasal transactional morphology may reflect its pump mechanism, with duct contractions altering lumen shape according to physiological state at fixation [61,65,66].

#### 4.2.7. Ear

In rats and mice, the external ear canal is short and relatively straight [51,53]. SHs show a similar canal structure on transverse MCT and sectional anatomy.

#### 4.2.8. Eye

The orbit of SHs is generally ovoid, as seen on MCT and sectional anatomy, similar to mice and rats [51,53], whereas guinea pigs have a more spherical orbit [22,55]. The dorsal part of the orbit of SHs is largely unprotected by bone, as demonstrated by 3D MCT and with dried skulls, similar to mice, rats, and rabbits [18,48,51]; in contrast, cavies lack bony protection in the caudoventral orbital region [22,55]. A distinctive feature of SH eyes is a very large, nearly spherical lens occupying much of the intraocular space on MRI, resembling that of rats and mice, while rabbits possess a smaller lens without this pronounced spherical shape [5,67].

The harderian gland, recognized as the largest ocular gland in many mammals, including rabbits, and also termed the accessory lacrimal gland [67,68], shows interspecies variation in size and position [46,68,69]. In SHs, this large, lobulated gland occupies much of the ventromedial and posterior orbit, as confirmed by sectional anatomy and MRI, similar to rabbits, mice, and rats [5,46,51,67,70], whereas in cavies, the smooth gland is nearly as large as the eyeball and envelops it ventrally [5,67]. Additionally, the harderian gland in SHs exhibits sexual dimorphism; in females, it appears only as dark spots on gross anatomy, as previously reported [71], a feature not observed in Siberian (*Djungarian*) hamsters, other rodents, or rabbits [5,46,67]. Furthermore, unlike rabbits [19], the subcutaneous extraorbital lacrimal gland of SHs is positioned between the eye and ear pinna over the masseter muscle, a location confirmed by gross anatomy, MRI, and sectional anatomy and comparable to that reported in rats, mice, and cavies [46,51,53].

From a comparative morphological perspective, the anatomical features of SH heads are largely consistent with those of mice and rats—except for the presence of cheek pouches—but differ markedly from those of guinea pigs and rabbits.

Clinically, these findings provide valuable anatomical information to support the diagnosis, treatment, and surgical management of cheek pouch disorders (e.g., impaction, eversion, prolapse, abscesses, fistulae, and neoplasia); dental and oral conditions (including incisor malocclusion, caries, periodontal disease, facial or masseter abscesses, mandibular fractures, and symphyseal separation); and diseases or procedures involving the cervical region, upper respiratory tract, eyes, and ears. These include subcutaneous injections, nasal sampling, ear canal ablation with pinnectomy, and the management of exophthalmos, panophthalmitis, proptosis, otitis externa or media, as well as ear abscesses or neoplasia [4,50,52,72,73,74,75].

Head vessels seen in gross anatomical slices were not clearly discernible on the corresponding MR or micro-CT images, owing to limited soft-tissue contrast, a limitation also noted in rabbits [18,19,48]. To improve the identification of head vascular structures and aid in the precise localization of visceral anatomy in Syrian hamsters, dedicated vascular imaging techniques such as CT or MR angiography with intravenous contrast medium would be advantageous, as mentioned in rabbit studies [18,19,48]. In addition, latex vascular injection allows precise assessment of vascular anatomy and represents a useful adjunct for the interpretation of planimetric imaging modalities. Accordingly, because the primary aim of this study was to document baseline micro-CT and MRI images acquired through standard procedures, intravenous administration of contrast medium was not employed. It should be noted that, although most studies use high–atomic-number contrast agents to enhance the inherently low soft-tissue contrast, applying contrast-enhanced micro-CT in small living animals remains challenging due to the rapid renal clearance of these agents compared with humans [76]. Another limiting factor was the lack of histopathological confirmation of the normalcy of tissues.

## 5. Conclusions

Clinically important head structures seen in transverse, dorsal, and sagittal anatomical sections could be correspondingly recognized on micro-CT and/or MRI images. In Syrian hamsters, head MCT is particularly effective for visualizing mineralized structures (e.g., dental and osseous tissues) and air-filled cavities (e.g., the ear canal and tympanic bulla), whereas MRI provides superior assessment of soft tissues, including the brain, spinal cord, fluids, masticatory muscles, cheek pouches, intervertebral disks, parotid and mandibular salivary glands, and the eyeball’s structures (vitreous humor and lens, as well as harderian and extraorbital lacrimal glands). The present work provides a descriptive and imaging-based anatomical reference of the SH head, integrating anatomical sections, in situ topographical anatomy, and dry-skull photographs with micro-CT and MRI datasets, serving as a foundational resource for the interpretation of cross-sectional imaging in both research and clinical contexts.

## Data Availability

The original contributions presented in the study are included in the article; further inquiries can be directed to the corresponding author.

## Abbreviations

The following abbreviations are used in this manuscript:

micro-CT or MCT	Micro-computed tomography
MRI	Magnetic resonance imaging
SHs	Syrian hamsters

## References

[1] Suckow M.A., Stevens K.A., Wilson R.P. (2011). The Laboratory Rabbit, Guinea Pig, Hamster, and Other Rodents.

[2] Krautwald-Junghanns M.-E., Pees M., Reese S., Tully T. (2010). Diagnostic Imaging of Exotic Pets: Birds, Small Mammals, Reptiles.

[3] Capello V. (2016). Diagnostic imaging of dental disease in pet rabbits and rodents. Vet. Clin. N. Am. Exot. Anim. Pract..

[4] Quesenberry K., Mans C., Orcutt C. (2020). Ferrets, Rabbits, and Rodents: Clinical Medicine and Surgery.

[5] Gelatt K.N., Ben-Shlomo G., Gilger B.C., Hendrix D.V.H., Kern T.J., Plummer C.E. (2021). Veterinary Ophthalmology.

[6] Głodek J., Adamiak Z., Przeworski A. (2016). Magnetic resonance imaging of reptiles, rodents, and lagomorphs for clinical diagnosis and animal research. Comp. Med..

[7] Schwarz T., Saunders J. (2011). Veterinary Computed Tomography.

[8] Del Chicca F., Puccinelli C., Petrini D., Citi S. (2023). Incidental findings in computed tomography examination of the head in rabbits and guinea pigs. Vet. Sci..

[9] Li H., Zhang H., Tang Z., Hu G. (2008). Micro-computed tomography for small animal imaging: Technological details. Prog. Nat. Sci. Mater. Int..

[10] Rosenhain S., Magnuska Z., Yamoah G., Al Rawashdeh W., Kiessling F., Gremse F. (2018). A Preclinical Micro-Computed Tomography Database Including 3D Whole Body Organ Segmentations. Sci. Data.

[11] Seo J., Brown M. (2020). Experimental Animal Models for Meniere’s Disease: A Mini-Review. J. Audiol. Otol..

[12] Mai W. (2018). Diagnostic MRI in Dogs and Cats.

[13] Gavin P.R., Holmes S.P. (2009). Practical Small Animal MRI..

[14] Lee J.H., Park K., Kang T.C., Choung Y.H. (2009). Three-dimensional anatomy of the temporal bone in normal mice. Anat. Histol. Embryol..

[15] Yamada K., Chen C.J., Satoh H., Hirota T., Aoyagi K., Enkawa T., Ozaki Y., Sekiguchi F., Furuhama K. (1997). Magnetic Resonance Imaging of Rat Head with a High-Strength (4.7 T) Magnetic Field. J. Vet. Med. Sci..

[16] Denic A., Macura S.I., Mishra P., Gamez J.D., Rodriguez M., Pirko I. (2011). MRI in rodent models of brain disorders. Neurotherapeutics.

[17] Theunissen E., Baeten K., Vanormelingen L., Lambrichts I., Beuls E., Gelan J., Adriaensens P. (2010). Detailed Visualization of the Functional Regions of the Rat Pituitary Gland by High-Resolution T2-Weighted MRI. Anat. Histol. Embryol..

[18] Van Caelenberg A.I., De Rycke L.M., Hermans K., Verhaert L., van Bree H.J., Gielen I.M. (2010). Computed Tomography and Cross-Sectional Anatomy of the Head in Healthy Rabbits. Am. J. Vet. Res..

[19] Van Caelenberg A.I., De Rycke L.M., Hermans K., Verhaert L., van Bree H.J., Gielen I.M. (2011). Low-Field Magnetic Resonance Imaging and Cross-Sectional Anatomy of the Rabbit Head. Vet. J..

[20] Morales-Bordon D., Encinoso M., Arencibia A., Jáber J.R. (2023). Cranial Investigations of Crested Porcupine (*Hystrix cristata*) by Anatomical Cross-Sections and Magnetic Resonance Imaging. Animals.

[21] Morales Bordon D., Suárez-Cabrera F., Ramírez G., Paz-Oliva P., Morales-Espino A., Arencibia A., Encinoso M., Ventura M.R., Jaber J.R. (2024). Study of the Normal Crested Porcupine (*Hystrix cristata*) Nasal Cavity and Paranasal Sinuses by Cross-Sectional Anatomy and Computed Tomography. Vet. Sci..

[22] Mahdy M.A.A. (2021). Correlation between Computed Tomography, Magnetic Resonance Imaging and Cross-Sectional Anatomy of the Head of the Guinea Pig (*Cavia porcellus*, *Linnaeus* 1758). Anat. Histol. Embryol..

[23] Moselhy A.A., Mahdy E.A. (2019). Comparative Three-Dimensional Computed Tomography (CT) Scans and Anatomical Investigation of Rabbit (*Oryctolagus cuniculus*) and Cat (*Felis domestica*) Skull. Slov. Vet. Res..

[24] Müllhaupt D., Augsburger H., Schwarz A., Fischer G., Kircher P.R., Hatt J., Ohlerth S. (2015). Magnetic Resonance Imaging Anatomy of the Rabbit Brain at 3 T. Acta Vet. Scand..

[25] da Silva Alves L., Vulcano L.C., Girotto C.H., de Castro Sasahara T.H., Schimming B.C. (2024). Anatomy of the Brain of Capybara (*Hydrochoerus hydrochaeris*) Using Magnetic Resonance Imaging. Anat. Histol. Embryol..

[26] Phillips J.E., Ji L., Rivelli M.A., Chapman R.W., Corboz M.R. (2009). Three-Dimensional Analysis of Rodent Paranasal Sinus Cavities from X-ray Computed Tomography (CT) Scans. Can. J. Vet. Res..

[27] Brenner S.Z.G., Hawkins M.L., Tell L.A., Hornof W.J., Plopper C.G., Verstraete F.J.M. (2005). Clinical anatomy, radiography, and computed tomography of the chinchilla skull. Compend. Contin. Educ. Pract. Vet..

[28] Pereira F.M.A.M., Bete S.B.d.S., Inamassu L.R., Mamprim M.J., Schimming B.C. (2020). Anatomy of the Skull in the Capybara (*Hydrochoerus hydrochaeris*) Using Radiography and 3D Computed Tomography. Anat. Histol. Embryol..

[29] Jacob A., Chole R.A. (2006). Survey anatomy of the paranasal sinuses in the normal mouse. Laryngoscope.

[30] Van Spaendonck M.P., Cryns K., Van De Heyning P.H., Scheuermann D.W., Van Camp G., Timmermans J.P. (2000). High Resolution Imaging of the Mouse Inner Ear by Microtomography: A New Tool in Inner Ear Research. Anat. Rec..

[31] Smith T.D., Bonar C.J. (2022). The Nasal Cavity in Agoutis (*Dasyprocta* spp.): A Micro-Computed Tomographic and Histological Study. Vertebr. Zool..

[32] Chen K.C., Arad A., Song Z.M., Croaker D. (2018). High-definition neural visualization of rodent brain using micro-CT scanning and non-local-means processing. BMC Med. Imaging.

[33] Cox P.G., Jeffery N. (2011). Reviewing the morphology of the jaw-closing musculature in squirrels, rats, and guinea pigs with contrast-enhanced micro-CT. Anat. Rec..

[34] Nourinezhad J., Tabrizinejad M.N., Janeczek M. (2022). Detailed Gross Anatomy and Topography of the Sympathetic Cardiac Nerves and Related Ganglia in Syrian Hamsters (*Mesocricetus auratus*). Ann. Anat..

[35] Nourinezhad J., Ranjbar R., Rostamizadeh V., Tabrizinejad M.N., Hallak A., Janeczek M. (2023). Morphology of the Pattern of Branching of the Aortic Arch (Arcus Aortae) in Syrian Hamsters (*Mesocricetus auratus*). Vet. Res. Commun..

[36] Nourinezhad J., Homayonnezhad Z., Moarabi A., Hanafi M.G., Janeczek M. (2025). Evaluation of Sectional Anatomic, Micro-Computed Tomographic, and Magnetic Resonance Imaging Features of the Thorax in Syrian Hamsters (*Mesocricetus auratus*). Vet. Res. Commun..

[37] Hallak A., Ranjbar R., Nourinezhad J. (2023). Anatomical study of arterial arrangement of the spinal cord in Syrian hamsters (*Mesocricetus auratus*). Anat. Sci. Int..

[38] Mohammadzadeh N., Nourinezhad J., Moarabi A., Janeczek M. (2025). Sectional Anatomy with Micro-Computed Tomography and Magnetic Resonance Imaging Correlation of the Middle and Caudal Abdominal Regions in the Syrian Hamster (*Mesocricetus auratus*). Animals.

[39] Ansari S., Nourinezhad J., Moarabi A., Hanafi M.G., Janeczek M. (2023). Sectional Anatomy, Micro-Computed Tomography, and Magnetic Resonance Imaging of the Syrian Hamsters (*Mesocricetus auratus*) Head. Proceedings of the 34th Congress of the European Association of Veterinary Anatomists.

[40] Gad S.C., Gupta R.C. (2014). Rodents Model for Toxicity Testing and Biomarkers. Biomarkers in Toxicology.

[41] Silverman S., Tell L. (2004). Radiology of Rodents, Rabbits, and Ferrets: An Atlas of Normal Anatomy and Positioning.

[42] Brown R.W., Cheng Y.N., Haacke E.M., Thompson M.R., Venkatesan R. (2014). Magnetic Resonance Imaging.

[43] Hildebrand M. (1968). Anatomical Preparations.

[44] Leary S.L., Underwood W., Anthony R., Cartner S., Corey D., Grandin T., Greenacre C., Gwaltney-Brant S., McCrackin M.A., Meyer R. (2013). AVMA Guidelines for the Euthanasia of Animals.

[45] Popesko P., Rajtová V., Horák J. (1992). A Colour Atlas of the Anatomy of Small Laboratory Animals, Volume Two: Rat, Mouse, Hamster.

[46] Smallwood J.E. (1992). A Guided Tour of Veterinary Anatomy: Domestic Ungulates and Laboratory Mammals.

[47] International Committee on Veterinary Gross Anatomical Nomenclature (2017). Nomina Anatomica Veterinaria.

[48] De Rycke L.M., Boone M.N., Van Caelenberg A.I., Dierick M., Van Hoorebeke L., van Bree H., Gielen I.M. (2012). Micro-Computed Tomography of the Head and Dentition in Cadavers of Clinically Normal Rabbits. Am. J. Vet. Res..

[49] Sasai H., Fujita D., Tagami Y., Seto E., Denda Y., Hamakita H., Ichihashi T., Okamura K., Furuya M., Tani H. (2015). Characteristics of Bone Fractures and Usefulness of Micro–Computed Tomography for Fracture Detection in Rabbits: 210 Cases (2007–2013). J. Am. Vet. Med. Assoc..

[50] Böhmer E. (2015). Dentistry in Rabbits and Rodents.

[51] Ruberte J., Carretero A., Navarro M. (2017). Morphological Mouse Phenotyping: Anatomy, Histology and Imaging.

[52] Mancinelli E., Capello V. (2016). Anatomy and Disorders of the Oral Cavity of Rat-like and Squirrel-like Rodents. Vet. Clin. N. Am. Exot. Anim. Pract..

[53] Hebel R., Stromberg M.W. (1976). Anatomy of the Laboratory Rat.

[54] Cook M.J. (1965). The Anatomy of the Laboratory Mouse.

[55] Cooper G., Schiller A.L. (1975). Anatomy of the Guinea Pig.

[56] Popesko P., Rajtová V., Horák J. (1992). A Colour Atlas of the Anatomy of Small Laboratory Animals, Volume One: Rabbit, Guineapig.

[57] Barone R., Pavaux C., Bline P.C., Cuq P. (1973). Atlas d’Anatomie du Lapin.

[58] Barone R. (1986). Anatomie Comparée des Mammifères Domestiques. Tome 1: Ostéologie.

[59] Donnelly T.M., Vella D. (2016). Anatomy, Physiology and Non-dental Disorders of the Mouth of Pet Rabbits. Vet. Clin. N. Am. Exot. Anim. Pract..

[60] Matosz B., Dezdrobitu C., Martonos C., Luca V., Bogdan S., Damian A. (2016). Major Salivary Glands Topography in Rats and Their Relation with the Surrounding Anatomical Tissues. Sci. Work. Ser. C Vet. Med..

[61] Dennis J.C., Stilwell N.K., Smith T.D., Park T.J., Bhatnagar K.P., Morrison E.E. (2020). Is the mole rat vomeronasal organ functional?. Anat. Rec..

[62] Baverstock H., Jeffery N.S., Cobb S.N. (2013). The Morphology of the Mouse Masticatory Musculature. J. Anat..

[63] Cox P.G., Watson P.J. (2023). Masticatory biomechanics of red and grey squirrels (*Sciurus vulgaris* and *Sciurus carolinensis*) modelled with multibody dynamics analysis. R. Soc. Open Sci..

[64] Taniguchi K., Mochizuki K. (1983). Comparative Morphological Studies on the Vomeronasal Organ in Rats, Mice, and Rabbits. Jpn. J. Vet. Sci..

[65] Villamayor P.R., Cifuentes J.M., Fdz-de-Troconiz P., Sanchez-Quinteiro P. (2018). Morphological and Immunohistochemical Study of the Rabbit Vomeronasal Organ. Animals.

[66] Vaccarezza O.L., Sepich L.N., Tramezzani J.H. (1981). The Vomeronasal Organ of the Rat. J. Anat..

[67] Eaton J.S., Montiani-Ferreira F., Moore B.A., Ben-Shlomo G. (2022). Ophthalmology of Myodonta: Mice, Rats, Hamsters, Gerbils, and Relatives. Wild and Exotic Animal Ophthalmology.

[68] Payne A.P. (1994). The Harderian gland: A tercentennial review. J. Anat..

[69] Hittmair K.M., Tichy A., Nell B. (2014). Ultrasonography of the Harderian gland in the rabbit, guinea pig, and chinchilla. Vet. Ophthalmol..

[70] Sbarbati A., Calderan L., Nicolato E., Marzola P., Lunati E., Benati D., Bernardi P., Osculati F. (2002). Magnetic resonance imaging of the rat Harderian gland. J. Anat..

[71] Christensen F., Dam H. (1953). Sexual dimorphism of the Harderian glands in hamsters. Acta Physiol. Scand..

[72] Capello V. (2003). Surgical techniques in pet hamsters. Exotic DVM.

[73] Bennett R.A., Pye G.W. (2022). Surgery of Exotic Animals.

[74] Capello V. (2011). Common surgical procedures in pet rodents. J. Exotic Pet Med..

[75] Martorell J.M., Soto Martín S., Martínez A. (2010). Complete ablation of the vertical auditory canal and ear pinna in a dwarf hamster (*Phodopus sungorus*) with spontaneous aural squamous cell carcinoma. J. Exotic Pet Med..

[76] Clark D.P., Badea C.T. (2021). Advances in micro-CT imaging of small animals. Phys. Med..

